# Examining the risk of delirium in patients hospitalized with COVID-19: Insights from the homeless population

**DOI:** 10.1371/journal.pone.0313242

**Published:** 2025-01-09

**Authors:** Liam O’Neill, Neale R. Chumbler

**Affiliations:** 1 Department of Rehabilitation and Health Services, College of Health and Public Service, University of North Texas, Denton, TX, United States of America; 2 Department of Diagnostic and Health Sciences, College of Health Professions, University of Tennessee Health Science Center, Memphis, TN, United States of America; Hospital Sirio-Libanes, BRAZIL

## Abstract

For patients hospitalized with COVID-19, delirium is a serious and under-recognized complication, and people experiencing homelessness (PEH) may be at greater risk. This retrospective cohort study compared delirium-associated risk factors and clinical outcomes between PEH and non-PEH. This study used patient records from 154 hospitals discharged from 2020–2021 from the Texas Inpatient Public Use Data file. Study subjects (n = 878) were patients, aged 18–69 years, who were hospitalized with COVID-19 and were identified as homeless. The baseline group included (n = 176,518) patients with COVID-19 aged 18–69 years who were not homeless. Logistic regression models were used to identify risk factors for delirium. Relevant risk factors included chronic comorbidities, substance use disorders, and traumatic brain injury (TBI). Seven of the delirium-associated risk factors were more prevalent among PEH compared to baseline. PEH had higher rates of TBI, alcohol, cannabis, and opioid use disorders. PEH had significantly higher rates of delirium (10.6% vs. 8.1%; P<0.01). However, PEH had fewer respiratory complications, including pneumonia (48.5% vs. 81.9%; P<0.001) and respiratory failure (28.7% vs. 61.9%; P<0.001), and lower in-hospital mortality (3.3% vs. 9.5%; P<0.001). The anti-viral Remdesivir had a protective effect against delirium (AOR = 0.63; CI: 0.60, 0.66). Mean hospital length of stay (LOS) was more than twice as long for delirious patients compared with non-delirious patients (18.4 days vs. 7.7 days; P<0.001). Delirium greatly increased the risk of in-hospital mortality (AOR = 3.8; CI: 3.6, 4.0). For PEH (n = 29) who died from COVID-19, delirium was present in more than half (52%) of cases. Hospitals should screen PEH for delirium and adopt nursing protocols to prevent delirium and reduce its severity.

## Introduction

Delirium is a neuropsychiatric condition that involves a sudden onset of cognitive deficits, particularly in attention. There is significant overlap in the literature between “delirium” and related terms, such as “acute confusion” and “acute encephalopathy” [1].

Following the onset of the pandemic in the spring of 2020, there was a surge in the number of hospitalized patients with delirium, regardless of their COVID-19 status [2]. The pandemic also led to significant disruptions in the daily routines of both hospitalized patients and staff. The relevant changes were caused by an increase in the demand for hospital resources, staff shortages, an increased use of benzodiazepines (sedatives), and rigorous infection control measures that led to social isolation and a lack of mobility [3]. This combination of factors is believed to be a major cause of the observed increase in delirium. A longitudinal study was conducted that included more than two million patients admitted to Canadian hospitals from 2017 to 2022. It found that the overall prevalence of delirium increased by about 15 percent at the start of the pandemic, and this affected all patients, regardless of their initial diagnosis [4]. This not only caused an increase in morbidity and mortality; it also exacerbated the shortage of life-saving hospital resources, such as staff time, ICU beds, and ventilators [2]. Early diagnosis of delirium is crucial, since it is a standalone predictor of increased risk of mortality and extended LOS. This is especially important in COVID-19 patients because delirium could be a warning sign for respiratory failure or an infection of the central nervous system [3].

Homelessness has been associated with a myriad of health concerns, including premature cognitive decline and accelerated aging [5, 6]. People experiencing homelessness (PEH) may have a higher prevalence of certain chronic comorbidities, which may increase their risk for neurological complications due to COVID-19, such as delirium. However, there are few studies on the impact of COVID-19 on PEH that include detailed clinical data, such as chronic comorbidities, substance use disorders (SUDs), hospital complications, and treatments, such as antiviral drugs.

Previous research has found that PEH are more likely to experience cognitive decline at a younger age than the general population. One study by Hurstak et al. (2017) found that PEH, who were mostly in their 50s, had significantly worse cognitive impairment than persons aged 70 and older [7]. Additionally, chronic alcohol use among PEH was associated with global cognitive impairment. The use of alcohol and other substances by PEH may also have been exacerbated by the pandemic, as some reported increased feelings of loneliness and isolation due to social distancing [8]. Another study of PEH by Gicas et al. (2020) found that traumatic brain injury (TBI) and long-term alcohol dependence were linked to a decline in verbal memory and cognitive functioning [9]. Head injuries, which were reported in 54% of subjects, were also associated with cognitive decline [10].

A recent systematic review of nine studies found that homelessness may be a risk factor for Alzheimer’s disease and related dementia [11]. The study found that PEH are at greater risk for psychiatric disorders, substance abuse, and traumatic injuries, and these factors also increase the risk of Alzheimer’s disease. However, it remains unclear if these psychiatric disorders preceded becoming homeless or whether homelessness exacerbated these underlying conditions. The authors also stressed the need for future studies using non-veteran homeless populations.

The risk of delirium for hospitalized patients increases with age, and this is why most research on delirium has focused on patients age 65 and older [12]. By contrast, the age distribution of PEH is skewed by premature mortality; hence the "older" cohort of PEH are mostly in their forties and fifties [13, 14]. Thus the impact of delirium on this vulnerable population may have been overlooked. This study seeks to address this gap in the literature. We hypothesize that PEH have a higher prevalence of certain chronic diseases and other risk factors that increases their susceptibility to delirium.

The main purpose of this study was to compare delirium-associated risk factors and clinical outcomes between PEH and non-PEH using a retrospective cohort design. Risk factors include chronic comorbidities, substance use disorders, and traumatic brain injury. A secondary aim was to assess the impact of delirium on mortality, length of stay (LOS), and cost. In addition, we examined whether treatment with the antiviral Remdesivir was associated with a lower risk of delirium and mortality. Understanding the susceptibility of PEH to various complications from COVID-19 is crucial for developing targeted interventions and harm reduction strategies for this vulnerable population.

## Materials and methods

The data for this study came from the 2020–2021 Texas Inpatient Public Use Data File (TIPUDF), an administrative data set maintained by the Texas Department of State Health Services. This database contained one principal and twenty-four secondary diagnosis codes for each discharge. This is a comprehensive database that includes 93% to 97% of all hospital discharges statewide. The database included about 3.5 million hospital discharge records from April 1, 2020 to June 30, 2021.

Patients who were hospitalized with COVID-19 were identified based on the International Classification of Diseases, Tenth Revision, Clinical Modification (ICD-10-CM) rubric, U07.1 (COVID-19, virus identified), for either the principal diagnosis or any of 24 secondary diagnosis codes. Consistent with previous studies, PEH were identified using the ICD10-CM code ‘Z59.0’ (homelessness) for either the primary or any of the secondary diagnosis codes. Ninety percent of PEH were younger than 70 years old. Study subjects (n = 878) were those hospitalized with COVID-19, aged 18–69 years, and who were identified as homeless in the patient’s medical record. The baseline, comparison group (n = 176,518) included all COVID-19 patients, aged 18–69, who were not identified as homeless.

Eight chronic health conditions were included from the 30 comorbidities in the Elixhauser comorbidity index [15]. These were selected based on literature identifying them as risk factors [16, 17]. These included the following: cardiac arrhythmias, dementia, diabetes with complications, hemiplegia, hepatitis, chronic kidney disease, liver disease, and traumatic brain injury (TBI). Other variables included: Alcohol Use Disorder (AUD), Cannabis Use Disorder (CUD), and Opioid Use Disorder (OUD). The Healthcare Cost and Utilization Project’s Clinical Classifications Software was used to obtain the most current definitions of these comorbidities.

Remdesivir is an antiviral medication that is used to treat Covid-19. Patients who were treated with Remdesivir were identified using the ICD-10-PCS rubric ‘XW033E5’ or ‘XW043E5’ for either the primary or any of the secondary procedure codes [18]. Payer type was based on both the patient’s primary and, if applicable, secondary source of payment. There were two payer categories: commercial insurance and Medicaid. Two sources of hospital admission were included: emergency department and transfer from a skilled nursing facility [17]. Patients were categorized by Hispanic ethnicity and then by race (Asian, Black, White, and other).

### Statistical analysis

Significant differences between the two groups were identified using chi-square tests for categorical variables and t-tests for ordinal variables. Logistic regression models were used to predict the likelihood of delirium. Predictor variables included patient characteristics (race/ethnicity, age, sex, source of admission and health insurance) and chronic comorbidities. A forward stepwise regression model was employed to select the most significant variables. Variables were entered into the model if their associated p-value was less than or equal to 0.05. All confidence intervals were at the 95 percent level. The risk of delirium was estimated using the predicted values from the logistic model.

A cost analysis was performed to estimate the additional cost per case for of delirium. Consistent with prior studies, the cost per case of delirium was based on the mean difference in length of stay (LOS) between patients with and without delirium. Estimates of the cost per patient day for both acute-care and intensive care were derived from the study by Ohsfeldt et al., (2020) [19]. OLS regression was used to predict the patient’s length of stay, and the predictor variables included all risk factors for delirium, along with an indicator for delirium. Patients were split into two groups, depending on whether they were treated in the ICU. A separate regression model was run for each patient group. All statistical analyses were performed using SPSS software version 28 (IBM, Armonk, NY, 2021). Since the dataset was de-identified, this study was determined to be *Not Human Subjects Research* by the Institutional Review Board (IRB # 24–357) of the University of North Texas. The IRB determined that informed consent was not needed because this was not human subjects research. The data were accessed between July, 2022 and September, 2024 for the purpose of this analysis.

## Results and discussion

PEH had significantly higher rates of delirium (10.6% vs. 8.1%; P< 0.01), as shown in Table 1. However, PEH had fewer respiratory complications, including pneumonia (48.5% vs. 81.9%; P<0.001) and respiratory failure (28.7% vs. 61.9%; P<0.001), and lower in-hospital mortality compared to the baseline group (3.3% vs. 9.5%; P<0.001). With respect to mean length of stay (LOS), there was no significant difference between the two groups (8.3 days vs. 8.6 days; P > 0.1).

**Table 1:** Patient characteristics of homeless patients (Aged 18–69) compared to baseline group of patients hospitalized with Covid-19.

		Non-Homeless	Homeless	
		Patients	Patients	
Patients		(n = 176,518)	(n = 878)	P-value
**Demographics**				
	**Age**	52.3	50.2	0.000
	**Female**	42.1%	15.5%	
	**Male**	52.1%	37.5%	
	**Missing Gender**	5.8%	47.0%	0.000
**Race/Ethnicity**				
	**Asian**	2.0%	0.3%	
	**Black**	14.9%	24.9%	
	**Hispanic**	40.3%	24.7%	
	**White, Non-Hisp.**	35.4%	43.9%	
	**Other race**	7.5%	6.2%	0.000
**Source of Admission**				
	**Emergency Dept.**	83.6%	90.6%	> 0.05
	**Skilled Nurse Fac.**	0.4%	0.3%	> 0.05
**Source of Payment**				
	**Commercial Ins.**	44.1%	9.1%	0.000
	**Medicaid**	12.9%	25.9%	0.000
**Comorbidities**				
	**Cardiac Arrhythmias**	9.3%	9.2%	> 0.05
	**Dementia**	0.4%	0.3%	> 0.05
	**Diabetes with Comp.**	31.5%	28.6%	> 0.05
	**Hemiplegia**	1.2%	1.6%	> 0.05
	**Hepatitis**	1.8%	11.5%	0.000
	**Kidney, Chronic**	15.6%	18.2%	0.034
	**Liver Disease**	6.3%	16.2%	0.000
	**Traumatic Brain Injury**	0.3%	1.3%	0.000
**Substances**				
	**Alcohol use disorder**	2.8%	18.5%	0.000
	**Cannabis UD**	0.9%	7.2%	0.000
	**Opioid UD**	0.7%	6.4%	0.000
**Other**				
	**Delerium**	8.1%	10.6%	0.007
	**In-Hospital Mortality**	9.5%	3.3%	0.000
	**Remdesivir**	26.7%	8.4%	0.000

As shown in Table 1, PEH had significantly higher rates of hepatitis, chronic kidney disease, liver disease and traumatic brain injury. The incidence of TBI was about four times higher for PEH compared with the baseline group (1.3% vs. 0.3%; P<0.001). PEH were more likely to have Medicaid insurance and less likely to have commercial insurance. PEH had significantly higher rates of alcohol, cannabis, and opioid use disorders. Twenty-nine percent of PEH had at least one substance use disorder (SUD). By contrast, only four percent of the baseline group had one or more SUDs. The prevalence of SUDs was six to nine times higher among PEH. To summarize, seven of the delirium-associated risk factors were more prevalent among PEH compared to baseline.

Only two characteristics of the homeless population were associated with a reduced risk of delirium. PEH were younger compared to the baseline group (50.2 vs. 52.3 years, P<0.01). Second, PEH had slightly higher emergency admissions and slightly lower admissions from a skilled nursing facility, although these differences were not significant. Treatment with Remdesivir was associated with a lower risk of delirium. However, PEH were less likely to be treated with Remdesivir (8.4% vs. 26.7%; P <0.01).

The most influential patient categories, in terms of their explanatory power, were chronic comorbidities, followed by source of payment, and substance use disorders (SUDs). As shown in Table 2, the most significant predictors of delirium were dementia (AOR = 3.09; CI: 2.58, 3.70, opioid use disorder (AOR = 2.77; CI: 2.39, 3.21), and traumatic brain injury (AOR = 2.66; CI: 2.06, 3.42). Transfer from a skilled nursing facility (AOR = 2.49; CI: 2.08, 2.97) was also a risk factor for delirium. As shown in Table 2, protective effects for delirium included treatment with Remdesivir (AOR = 0.63; CI: 0.6, 0.66), female gender (AOR = 0.91; CI: 0.88, 0.94), Hispanic ethnicity (AOR = 0.86; CI: 0.82, 0.89), commercial insurance (AOR = 0.83; CI: 0.79, 0.86), and emergency department admission (AOR = 0.65; CI: 0.62, 0.68). Based on the predicted values from the logistic model, PEH also had a higher risk of delirium compared to baseline (11.7% vs. 8.1%; P<0.001).

**Table 2:** Logistic regression model predicting risk of delirium for patients hospitalized with COVID-19.

		Odds	Lower	Upper
Patients (N = 177,396)		Ratio	Limit	Limit
**Demographics**				
	**Age/10**	1.39	1.36	1.41
	**Female**	0.91	0.88	0.94
**Race/Ethnicity**				
	**Black**	1.08	1.02	1.14
	**Hispanic**	0.86	0.82	0.89
**Source of Admission**				
	**Emergency Dept.**	0.65	0.62	0.68
	**Skilled Nurse Facility**	2.49	2.08	2.97
**Source of Payment**				
	**Commercial Ins.**	0.83	0.79	0.86
	**Medicaid**	1.58	1.50	1.66
**Comorbidities**				
	**Cardiac Arrhythmias**	1.74	1.66	1.83
	**Dementia**	3.09	2.58	3.70
	**Diabetes with Comp.**	1.43	1.37	1.48
	**Hemiplegia**	2.13	1.92	2.37
	**Hepatitis**	1.21	1.09	1.35
	**Kidney, Chronic**	1.82	1.74	1.90
	**Liver Disease**	1.10	1.02	1.18
	**Traumatic Brain Injury**	2.66	2.06	3.42
**Substances**				
	**Alcohol use disorder**	2.39	2.19	2.60
	**Cannabis use disorder**	1.54	1.29	1.83
	**Opioid use disorder**	2.77	2.39	3.21
**Other**				
	**Remdesivir**	0.63	0.60	0.66

Compared to patients with only one risk factor, patients with two risk factors had about twice the risk of delirium (16%). The risk of delirium was 21% for persons with three or more risk factors. Regardless of housing status, delirium was associated with significant morbidity and mortality. Mean length of stay (LOS) was about ten days longer for delirious patients compared with non-delirious patients (18.4 days vs. 7.7 days; P<0.001). For PEH, the average LOS was five days longer for delirious patients compared with non-delirious patients (12.9 days vs. 7.7 days; P<0.001). Based on the regression results, for ICU patients, having delirium increased their LOS by 9.5 days (CI: 9.3, 9.7). For acute-care patients, having delirium increased their LOS by 5.3 days (CI: 5.1, 5.5). In addition, eight percent of delirious patients required mechanical ventilation. More than one-fourth (28.5%) of delirious patients died in the hospital compared to 7.8% of non-delirious patients. Delirium was also found to greatly increase the risk of mortality (AOR = 3.8; CI = 3.6, 4.0). The additional cost per case of delirium was estimated to be $18,177 (CI: $16,872, $19,702).

Delirium is a well-known complication of respiratory disease among older adults. The causes of delirium are multifactorial, and these can be categorized as predisposing, precipitating, and environmental [1]. Predisposing factors include age, chronic medical conditions, and preexisting cognitive impairment. Precipitating factors include the acute illness itself, substance use and withdrawal, and the use of certain medications, such as benzodiazepines. Environmental effects, such as isolation and lack of cognitive stimulation, can also play a role [3]. This is the first study to find that PEH who were hospitalized with COVID-19 had higher rates of delirium compared to the baseline group.

PEH had lower rates of respiratory complications and in-hospital mortality, and these findings are consistent with a prior study from the Center for Disease Control (CDC) that found similar results [20]. We sought to better understand these findings. Obesity was found to be a major risk factor for pneumonia among COVID-19 patients [21]. With respect to the present study, obesity rates were more than twice as high in the baseline group (38.2%) compared to PEH (15.7%; P<0.001), where obesity was defined as a Body Mass Index (BMI) of 30 or greater. Because PEH had significantly lower rates of obesity, this was likely a contributing factor to their lower rates of respiratory complications, such as pneumonia and respiratory failure.

Among patients with delirium, PEH were significantly younger compared to baseline, as measured by median age (55 vs. 62 years; P<0.001.) Ostensibly, younger patients should have a reduced risk of delirium. However, the age distribution of the PEH in this study may reflect both accelerated aging and premature mortality. Previous studies have found homelessness to be associated with a shorter lifespan. For younger PEH, drug overdose was found to be the leading cause of death [13]. By contrast, older PEH, i.e., those in their forties and fifties, die from similar causes as the housed population; however, they die 10–15 years earlier [14]. Another study of 615 PEH found the average age at death to be 56 for males and 52 for females [22].

A recent systematic review found that older PEH are susceptible to accelerated aging and geriatric conditions, such as frailty, cognitive impairment, and functional decline [5]. Another study found that PEH had accelerated physical limitations, balance impairments, frailty, and lack of mobility, regardless of their age [23]. With respect to the baseline group, dementia (AOR = 3.09; CI: 2.58, 3.7) was found to be the most significant risk factor for delirium, as shown in Table 2. However, this was not the case for PEH. Among this group, the most significant risk factors were traumatic brain injury (TBI) (AOR = 6.55; CI: 1.85, 23.24) and AUD (AOR = 1.74; CI: 1.04, 2.90). AUD, as consistent with prior literature, was also associated with an increased risk of liver disease and hepatitis. In addition, PEH were more likely to have a diagnosis of chronic pain than baseline (0.7% vs. 0.1%; P < 0.001). Chronic pain has also been associated with an increased risk of delirium [1].

A recent systematic review and meta-analysis of 66 studies emphasized that delirium is a frequent complication of COVID-19 in older adults. However, it did not address whether PEH are at increased risk for delirium [24]. Since most of these studies focused on patients aged 65 and older, most PEH were likely excluded from the analyses. In the current study, approximately 80 percent (n = 774) of the PEH in the database (n = 974) were younger than 65 years.

Due to various factors, the onset of the pandemic coincided with an increase in SUDs and smoking, especially among vulnerable populations, such as PEH [8, 25]. Nyamathi et al., (2022), conducted in-depth interviews with 21 PEH to identify the reasons for the increase in SUDs [8]. The pandemic and subsequent restrictions increased feelings of isolation, anxiety, and depression. Some PEH used alcohol and smoking as a coping strategy, which were easy to obtain and were perceived as a "safe alternative" to street drugs, such as opioids. There were also cutbacks and reduced staffing for social services, such as mental health services, drug treatment programs, and needle exchange programs [26]. As only about ten percent of this population have cell phones, most PEH were unable to access telehealth services. Among the PEH in our study who had AUD (n = 162), about half (51%) were smokers. In addition, the percentage of smokers among PEH increased from 32% at the onset of the pandemic to 46% by June, 2021. It is likely that the true prevalence of smokers in this population was even higher [25].

Regardless of a patient’s housing status, delirium was associated with significant morbidity and mortality. Mean length of stay (LOS) was about ten days longer for delirious patients compared with non-delirious patients (18.4 days vs. 7.7 days; P<0.001). In addition, eight percent of delirious patients required mechanical ventilation. The in-hospital mortality rate for persons with delirium was 28.5% compared to 7.8% for non-delirious patients (P < 0.001). A separate logistic model was used to estimate the impact of delirium on in-hospital mortality. The mortality risk was 3.8 times higher (CI = 3.6, 4.0) for delirious patients compared to non-delirious patients.

Remdesivir was found to have a protective effect against delirium. This is consistent with prior research which found that the risk of in-hospital mortality was reduced by 17 percent for patients treated with Remdesivir [18]. Remdesivir was also found to have a protective effect against mortality (AOR = 0.68; CI: 0.65, 0.71). Patients with commercial insurance (P < 0.001) were more likely to be treated with Remdesivir. Those with Medicaid insurance, charity care, and substance use disorders (P < 0.001) were less likely to receive the treatment. We calculated mortality risk based on the risk factors in Table 2, excluding Remdesivir. Patients who received the drug had a lower *a priori* risk of mortality compared to those who did not receive it (8.6% vs. 9.7%; P < 0.001). Therefore, the observed differences in mortality may be partly due to differences in the risk profile of each group.

For PEH (n = 878), the risk of mortality was more than eight times higher for delirious patients as compared to those without delirium (AOR = 8.4; CI: 3.7–18.7). For PEH (n = 29) who died from COVID-19, delirium was present in more than half (52%) of cases. Hence delirium was found to greatly increase the risk of mortality for this vulnerable population. For delirious patients (n = 14,410), there was no significant difference in mortality between those who did or did not receive Remdesivir (28.9% vs. 28.4%; P > 0.05).

During the pandemic, many hospitals implemented infection control measures, such as isolation precautions, restrictions on family visitation, and limited physical contact with hospital staff. These measures often led to social isolation, lack of mobility, and even the use of physical and chemical restraints [3]. These environmental conditions may have exacerbated the risk of delirium and prolonged its severity and duration. A recent study of more than 33,000 hospitalized patients found that visitor restrictions, and the subsequent lack of human contact, imposed during the COVID-19 pandemic were associated with a 35 percent increase in the risk of delirium [27].

The incremental cost per case of delirium was estimated to be $18,177 (CI: $16,872, $19,702). This is somewhat consistent with prior literature [12]. During the period under study, the cost of the full course of treatment with the antiviral Remdesivir, received over five days, was about $3,100. Hence the savings that could be achieved by preventing a single case of delirium could pay for five patients to be treated with Remdesivir.

The most recent systematic review of the economic costs of delirium at the national level was based on multiple studies conducted before the pandemic. This review estimated that in 2019, the total cost of delirium at US hospitals could be up to $82.4 billion [12]. The corresponding incremental cost of delirium per hospital stay was estimated to be between $1,529 and $19,050. Ample evidence indicates that delirium increases costs across all settings, such as hospitals, long-term care facilities, and home care. Delirium is also a cause of dementia, and the cost of long-term care for persons with dementia is even higher [28]. The true costs to society are likely to be significantly more than these estimates because they do not include opportunity costs. Because patients with delirium had a longer LOS and treatment intensity, this also increased the demand for critical hospital resources, such as ventilators, ICU beds, and nursing staff. By greatly increasing hospital costs and resource requirements, delirium also put additional financial strain on hospitals [29]. Additional opportunity costs include years of lost productivity by the young elderly who are unable to work and must be institutionalized [28].

Among the strengths of this study, we examined homelessness as a potential risk factor for delirium and mortality for patients hospitalized with COVID-19. We also demonstrated that several risk factors for delirium are more prevalent among this vulnerable population. We performed multivariable analysis that included several potential confounders. The study also estimated the incremental cost of delirium based on changes in patients’ mean LOS. This study underscores that delirium leads to increased mortality and morbidity, a significantly longer LOS, and higher hospital costs. In addition, these findings suggest that the antiviral Remdesivir was effective in reducing the risk of delirium.

This study is subject to some limitations. Data on the patients’ housing status is likely to be missing from medical records. Hence the prevalence of PEH who have COVID-19 is likely to be underestimated, especially in the early stages of the pandemic when testing was limited. Because PEH may have limited access to both primary care and specialty care, their true prevalence of chronic conditions may be underdiagnosed. Hence these study findings may not be generalizable to the broader population of PEH who are hospitalized with COVID-19. We did not have information on the length of time a person was homeless. Moreover, delirium is often under-documented in administrative databases [30].

Long-term effects of delirium are not uncommon among patients who survive and are discharged from the hospital.^1^ According to previous studies, twenty percent of patients still show some symptoms of delirium six months after hospital discharge. For patients hospitalized with COVID-19, delirium has been associated with long-term cognitive decline and increased risk of dementia [4, 28].

## Conclusions

Hospitals can adopt various best practices and nursing protocols to prevent delirium [2, 31]. An example of such a prevention program could be based on the following guidelines: screen for delirium; establish sleep routines; promote safe mobility; prioritize daily rounds; improve patient-family communication; boost cognitive engagement; and minimize restraints. Finally, pharmacological management of delirium should only be adopted in the most challenging cases [2, 31]. Such programs have proven to be cost-effective in reducing both delirium and related health care costs [32, 33]. Moreover, it is estimated that the implementation of such methods could prevent up to 40 percent of delirium cases or minimize their severity [27].

In the aftermath of the pandemic, delirium rates have remained elevated due to various factors, compared to pre-pandemic levels [4, 34]. Moreover, delirium remains under-recognized by healthcare professionals and underdiagnosed in hospitals [35]. According to one recent study, this is partly due to insufficient staff training regarding delirium screening and prevention [34]. To address this important issue, hospitals should screen PEH for delirium and identify those most at risk, so that it can be diagnosed and treated early in the patient’s clinical trajectory.
